# Adaptive divergence in diets between the sexes in a tropical snake (*Stegonotus australis*, Colubridae)

**DOI:** 10.1007/s00442-025-05689-1

**Published:** 2025-03-05

**Authors:** Gregory P. Brown, Thomas Madsen, Richard Shine

**Affiliations:** 1 School of Natural Sciences, Macquarie University, Sydney, NSW 2109 Australia; 2 School of Life and Environmental Sciences, Deakin University, Victoria, 3217 Australia

**Keywords:** Oophagy, Sexual dimorphism, *Stegonotus cucullatus*, Trophic niche, *Tropidonophis mairii*

## Abstract

Males and females within a population may differ in dietary composition either as a non-adaptive consequence of sexual dimorphism, or because specific food types enhance fitness more in one sex than the other. To test between those two explanations, we can ask whether the consumption of a food type (a) is constrained by sexually dimorphic traits such as body size, or (b) differentially benefits the sex that consumes that food more frequently. A 23-year field study of Slatey-Grey Snakes (*Stegonotus australis*) in tropical Australia provided data on 663 meals, of which 130 were reptile eggs (primarily from Keelback Snakes (*Tropidonophis mairii*)). Over the same range of snake SVLs, eggs were consumed more often by female than by male Slatey-Grey Snakes (25.8 versus 15.2% of records), but consumption of reptile eggs was independent of snake body size. Female Slatey-Grey Snakes were not more common or more active than males during Keelback nesting periods, but they were more likely to be captured in the vicinity of Keelback oviposition sites than were males. In years with higher availability of Keelback eggs, female Slatey-Grey Snakes had higher clutch sizes and clutch masses (plausibly reflecting the nutritional benefits of eating reptile eggs to provision reptile eggs). In combination, our results suggest an adaptive basis to the sex-based divergence in dietary composition in Slatey-Grey Snakes.

**Supplementary Information:**

The online version contains supplementary material available at 10.1007/s00442-025-05689-1.

## Introduction

In many animal species, adult males and females differ not only in morphological traits such as body sizes and body shapes, but also in ecological traits such as habitat use and dietary composition (Beck et al. 2007; Han et al. 2020; Rosalino et al. 2009; Shine et al. 1998; Tesler et al. 2019). In extreme cases, there is no overlap in the types of food consumed by males and females. For example, adult female mosquitoes extract blood from vertebrates, whereas adult males do not (Ignell et al. 2010; Shine 1989). In many other taxa, diets overlap partly but not completely between the sexes. For example, in gape-limited predators with extreme sexual size dimorphism (SSD), the larger sex often ingests larger prey items than does the smaller sex (Pearson et al. 2002).

The evolution of sex-based dietary divergence has been attributed to two different processes, one non-adaptive and one adaptive. The non-adaptive explanation interprets sex differences as secondary consequences of sexual dimorphism in other traits (e.g. body size or shape, coloration, behaviour) that themselves have been driven by sexual selection or fecundity selection. For example, males may grow larger than females if reproductive success in males depends upon victory in male–male combat bouts (Shine 1994). At the other extreme, females may attain larger sizes than males if fecundity is limited by maternal body size (e.g. Hendry et al. 2014). Likewise, male–male combat may favour the elaboration of jaw musculature, that in turn allows a male to subdue and ingest larger prey (Herrel et al. 1996). If gape size constrains maximal prey size, extreme sexual size dimorphism (SSD) is likely to generate sex divergences in prey types. Under this hypothesis, sex-based differences in dietary composition are non-adaptive consequences of morphological dimorphism. The alternative explanation is that specific food types enhance fitness in one sex more than the other (e.g. Houslay et al. 2015; Reddiex et al. 2013). This hypothesis fits well with the female’s need for a blood meal to fuel egg production in mosquitoes and other blood-sucking insects (see Shine 1989 for a review), as well as a trend for females of many mammalian and avian species to select more nutrient-rich, easily digestible food sources than do males (Beier 1987; Du Toit 2005; Raubenheimer and Simpson 2018). Although correlations between SSD and dietary divergence have been used to support the “non-adaptive” hypothesis (Bauld et al. 2022), adaptive factors may play a role as well: for example, larger body size may render one sex more capable of ingesting and digesting a particular type of food (Bauld et al. 2022). Hence, it is difficult to distinguish between adaptive and non-adaptive interpretations of sex-based dietary divergence without detailed ecological data. Ideally, we need information not only on the link between body size and diet within a single population, but also on the possibility that eating a specific prey type benefits individuals of one sex more than the other. Our long-term mark-recapture study of snakes in tropical Australia provide such data.

**Fig. 1:**
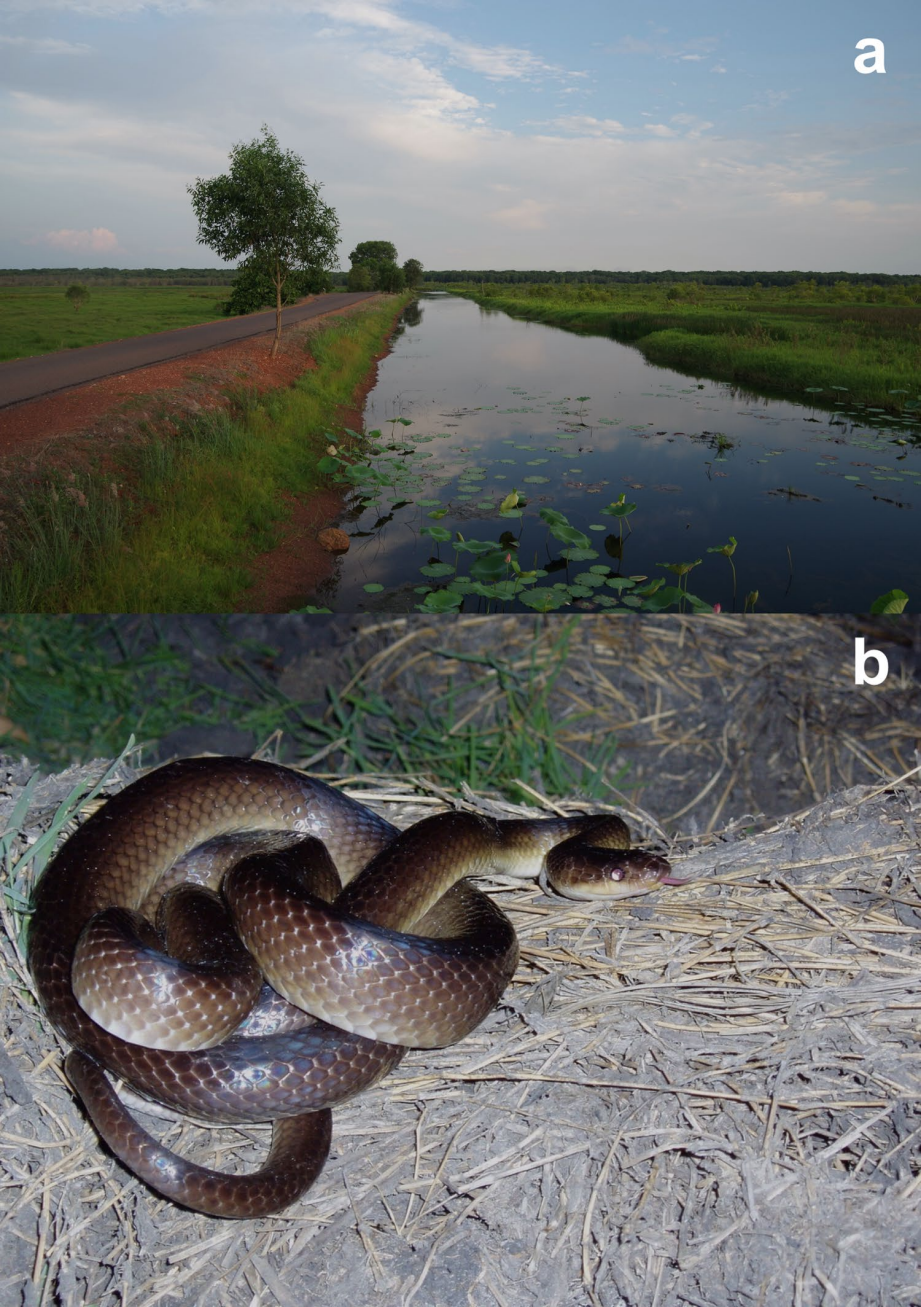
Our study site, the wall of Fogg Dam (**a**) and a female Slatey-Grey Snake, *Stegonotus australis,* at Fogg Dam (**b**)

Slatey-Grey Snakes (*Stegonotus australis,* formerly *S. cucullatus*: Kaiser et al. 2018) are muscular colubrids active in both arboreal and terrestrial habitats (Fig. 1). Slatey-Grey Snakes are sexually dimorphic in adult body size, with average snout-to-vent lengths (SVLs) of 1160 mm in males and 980 mm in females (Brown et al. 2017). In our study area, we have recorded females nesting over an 8-month period, from the late dry season (August) to the late wet season (March). Females can produce 1 to 2 clutches of 5 to 20 eggs annually, with larger clutches in larger females (Brown et al. 2017). The minimum interval we have recorded between successive clutches is 77 days. We have observed social interactions (courting, mating, combat) on nine occasions over the study, all during August–October, the early months of the nesting season. Brown et al. (2017) reported, based on stomach contents and regurgitated prey items, that these snakes feed on a wide variety of vertebrate prey including frogs (31% of items), mammals (29%), fishes (4%), reptiles (21%) and reptile eggs (15%). Our study species is an Australian offshoot of a diverse colubrid lineage characterised by enlarged blade-like teeth at the rear of the mouth; these teeth are used to slit the leathery shells of reptile eggs (Coleman et al. 1993; Huang et al. 2011).

Our long-term studies on Slatey-Grey nakes enable us to ask the following questions regarding dietary divergence:

1. Do diets differ between the sexes?
2. Is dietary divergence a by-product of sexual size dimorphism?
3. Is dietary divergence related to differential foraging or activity patterns in males and females?
4. Does consumption of the differentially consumed prey type benefit one sex more than the other?

## Materials and methods

*Study site* Fogg Dam (12.56° S, 131.27° E; Fig. 1) is a 300 ha artificial water body located 50 km east of the city of Darwin in Australia’s Northern Territory. It is impounded by a man-made 1500-m-long earthen wall with a road running along the top. The area experiences a wet–dry tropical climate with consistently hot and seasonally wet conditions. Maximum daily air temperatures average > 31 °C year-round, but > 95% of the 1422 mm annual rainfall occurs during a 6-month “wet season” (November–May).

*Surveys of snake reproduction* On 6511 nights between May 1998 and October 2021, one of us walked or drove the length of the Fogg Dam wall soon after dusk. Snakes located by spotlight were captured by hand, placed into individual cloth bags and returned to our nearby laboratory for processing the next day. For our study purposes, we have divided the 1500 m length of the dam wall into 31 50-m sections. This enables us to record the location of each snake capture with some degree of accuracy. For each captured snake, we recorded its capture location, sex, SVL, lower jaw (mandible) length, and mass and gave it an individual mark by scale-clipping. We acquired information on the snakes’ diets by three means. First, some snakes were in the process of subduing or swallowing prey when they were captured, and we recorded the type of prey. Second, snakes occasionally regurgitated prey items whilst held in captivity overnight. These items were identified and recorded. Third, the majority of our diet data were obtained from snakes defecating during handling and abdominal palpation. We noted if the faeces contained either reptile eggshells, mammal hairs or unidentified ‘other’. The latter category is likely to consist largely of frogs, which Slatey-Grey Snakes are regularly seen eating and which are abundant in our study site (Brown and Shine 2016). Anurans do not leave detectable remains in faecal samples. In cases where multiple prey types were recovered from an individual, all were recorded. We treated each observation, regurgitation or faecal record from a given snake as a single data point (e.g. we did not count individual eggs in a faecal sample as independent data points, because they all likely came from a single clutch being ingested). In the current paper, we use data from 3100 captures of 1199 individually marked Slatey-Grey Snakes.

If we palpated shelled eggs in the oviducts of female Slatey-Grey Snakes, we held the individuals in captivity until they oviposited (median time in captivity = 7 days, range 0–42, 25–75% quartiles = 3–12 days). After laying, the females were reweighed and released back at their capture location.

### Surveys of prey availability

**Fig. 2:**
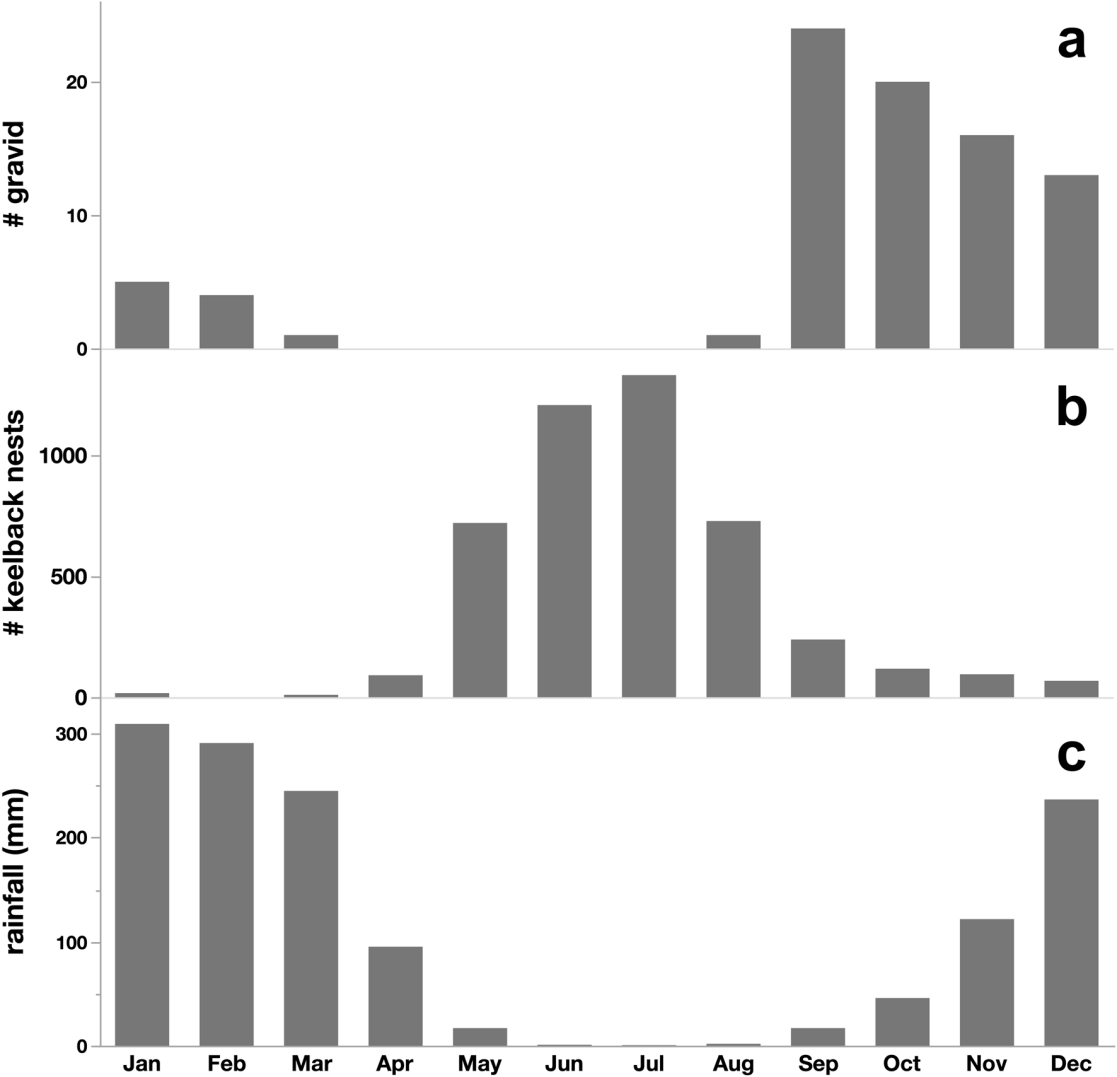
Monthly captures of gravid female Slatey-Grey Snakes at Fogg Dam summed over 23 years (**a**). Monthly presence of Keelback nests at Fogg Dam summed over 23 years, based on captures of gravid female Keelbacks and a 60-day incubation period (**b**). Mean monthly rainfall illustrating wet and dry seasonality (**c**)

1. *Reptile eggs* Slatey-Grey Snakes likely prey upon the eggs of several species of lizards and snakes, but those of Keelback Snakes (*Tropidonophis mairii*) appear to be the most important (based on the size of eggshells and their seasonal occurrence in faeces: see Results). Gravid female Keelbacks migrate to Fogg Dam between March and November to nest. Keelback eggs incubate for approximately 60 days. Thus, the peak period when Keelback eggs are at Fogg Dam is between May and August—the 3-month period prior to Slatey-Grey Snake’s nesting period, when females of the latter species are allocating energy to their developing clutches of eggs (Fig. 2); (see Brown et al. 2002, 2017). We use counts of gravid Keelbacks during March–August each year as an index of availability of reptile eggs for foraging Slatey-Grey Snakes as they undergo vitellogenesis. For the 20 years analysed in the present study, annual counts of gravid Keelbacks ranged from 5 to 190 (mean = 62, SE = 12.1). Much of the annual variation in population structure and reproductive measures in these and other local ophidian predators is driven by fluctuations in wet-season rainfall (Brown and Shine 2007; Madsen et al. 2006; Ujvari et al. 2016).
2. *Frogs* Beginning in 1999, we incorporated counts of native frogs into our nightly snake surveys. We painted ten 2 × 3 m survey grids, spaced 150 m apart, onto the surface of the road atop the wall of Fogg Dam. Each night we identified and counted frogs inside each grid during our final trip across the dam wall. We counted frogs in each grid a single time each night to avoid the possibility of counting the same individual on multiple occasions per night. We used the combined abundance of all frog taxa each night as an index of frog availability in our analyses, but four species (*Litoria bicolor*, *L. dahlii*, *L. nasuta* and *L. rothii*) comprised 94% of frogs encountered in our surveys (Brown and Shine 2016). We calculated average nightly frog counts during March–August each year, to correspond to the same period when we assessed numbers of gravid female Keelbacks (see above), 6 months prior to Slatey-Grey Snake nesting. For the 20 years analysed in the present study, average nightly frog counts ranged from 0.19 to 2.44 (mean = 8.33, SE = 0.12).
3. *Rodents *Dusky rats (*Rattus colletti*) and Grassland Melomys (*Melomys burtoni*) are often abundant on the floodplain adjacent to Fogg Dam. We trapped them there each year from 1999 to 2020 (except for 2004 and 2006). Trapping was conducted over a 5-day period each year with 50 Elliott traps placed at 10-m intervals along a 500 m transect; trap placement was consistent across years. The annual trapping session was carried out during the late dry season (August–September) each year. Trapped rodents were individually marked with ear tags. For the 20 years analysed in the present study, annual total counts of Melomys and Dusky Rats ranged from 0 to 145 (mean = 48, SE = 10.0).

*4. Climate *Because weather conditions can influence rates of foraging and digestion in ectotherms, we incorporated air temperature and rainfall into our analyses, using data from Darwin Airport (50 km from our site). For the 20 years analysed in the present study, average air temperature ranged from 25.6 to 28.2 °C (mean = 26.9, SE = 0.14) and rainfall ranged from 139 to 731 mm (mean = 405, SE = 39.7).

### Analyses

To address our first two questions—whether dietary composition differed between male and female Slatey-Grey Snakes and whether body size differences were responsible for such a difference—we performed a nominal logistic regression on our three-level prey-type variable (mammal/eggs/other) with sex and SVL as the independent variables. Prey type was fitted as a non-ordered multinomial variable. Our initial analysis included prey from Slatey-Grey Snakes over the full range of body sizes, from 235 to 1550 mm SVL. We then repeated this analysis after limiting the body size range to that at which adult male and female Slatey-Grey Snakes overlapped (SVL 800–1230 mm), thus excluding juvenile snakes and males that were much larger than any female. Because several individual snakes contributed multiple faecal samples over the course of the study, we used snake ID as a repeated subject variable in the analyses. We used the Genmod procedure in SAS 9.4 (SAS Institute, Cary, NC) to carry out the analysis. We performed an ANCOVA to compare relative jaw lengths of male and female Slatey-Grey Snakes. Jaw length was the dependent variable in the analysis with sex as the independent variable and SVL as the covariate.

To assess whether the reptile eggs detected in Slatey-Grey Snake faeces were likely to be from Keelbacks, as opposed to some other egg-laying reptile, we used Pearson correlation tests on average monthly counts of gravid Keelbacks *versus* average monthly counts of Slatey-Grey Snake prey items consisting of reptile eggs. We limited the analysis to adult-sized Slatey-Grey Snakes (> 800 mm SVL) because juvenile snakes would be unable to swallow eggs as large as those of Keelbacks.

Between March and November in our study area, gravid Keelbacks migrate from the floodplain to higher drier ground on the dam wall to oviposit (Brown et al. 2017, 2002), whereas radio-tracked Slatey-Grey Snakes move widely across the dam wall and associated floodplain habitats (Brown et al. 2005). If female Slatey-Grey Snakes preferentially target Keelback eggs as a food source, we would expect higher capture rates of female Slatey-Grey Snakes (relative to males) during the times of year and in the places where we record high capture rates of gravid Keelbacks. To assess our third question, regarding differential foraging activity of male and female Slatey-Grey Snakes, we compared the sex ratio (# females / # females + # males) of Slatey-Grey Snakes captured to the proportion of adult female Keelbacks that were gravid when captured. First, to compare the seasonal timing of the two measures, we used monthly mean sex ratios of Slatey-Grey Snakes and monthly mean proportions of gravid female Keelbacks, averaged over the 23 years of the study. Second, to compare the spatial occurrences of the two measures, we calculated their mean values in each of the 31 50-m sections of the Fogg Dam wall. Again, we calculated means of the two measures based on annual values averaged across 23 years but limited the analysis to the months of March–August when the majority of Keelback nesting occurs. We make the assumption that gravid Keelbacks would be likely to nest in the vicinity of their capture location. Both analyses included only adult-sized Slatey-Grey Snakes (> 800 mm SVL, *N* = 2344 captures). We used simple linear regression to assess seasonal and spatial relationships. We regressed mean Slatey-Grey Snake sex ratios on mean proportion of Keelbacks that were gravid, based on average monthly values in the first case and average spatial grid values in the latter case. To address our fourth question, we assessed the effects of prey availability and temperature on reproductive traits, limiting the analyses to captures of adult female Slatey-Grey Snakes made during the August–March nesting season for Keelbacks. We combined counts of Dusky Rats and Grassland Melomys trapped each year as ‘total rodents’ and counts of different frog species as ‘total frogs’. We did not have count data for frogs in 1998 or for mammals in 2004 or 2006, and thus did not include these years in the analyses relating prey abundance to reproduction.

Because reproductive output in Slatey-Grey Snakes depends on maternal body size (Brown et al. 2017), we included maternal SVL as a covariate in each model. Average air temperature and cumulative rainfall over the 6 months prior to each nesting period were included as independent variables in all models. Snake ID was included as a random effect because some individuals were captured multiple times. We also included the breeding season year as a random effect to accommodate any other potential annual fluctuations in factors other than prey abundance, temperature and rainfall. To model reproductive status (yes *versus* no), we used a binary distribution with logit link function in the generalised linear mixed model. To model egg and clutch measures, we used normal distributions and identity link functions. To model female post-laying body mass, we used a lognormal distribution and identity link function. Generalised linear mixed-models were performed using Proc Glimmix in SAS 9.4 using Kenward–Roger df approximation and a first-order autoregressive error structure to reduce potential autocorrelation and non-independence between successive breeding events. We used an *α* level of 0.05 and examined residuals for violations of assumptions of normality and homogeneity of variance.

Correlations amongst the five independent variables in each model that indexed prey abundance and climate were generally weak. Only two of the ten pairwise comparisons were significant (temperature and rainfall were negatively related; Pearson *r* = − 0.44, *P* = 0.04) and the number of gravid Keelbacks was positively related to the number of small mammals (Pearson *r* = 0.46, *P* = 0.04).

Because we assessed post-laying body mass of reproductive Slatey-Grey Snakes in relation to recent prey availability and climatic conditions, we conducted a similar analysis of body mass of male Slatey-Grey Snakes caught during the breeding season to compare to the relationships in conspecific females. We limited this analysis to male snakes within the same size range as adult females (800-1230mm SVL). As with the female analysis, we used SVL as a covariate, and prey availability (numbers of frogs, mammals and gravid Keelbacks) and climate measures (temperature and rainfall) as independent variables. Snake ID and breeding season year were used as random effects and we used a lognormal distribution and identity link function for the model.

## Results

### Do diets differ between the sexes of Slatey-Grey Snakes?

**Fig. 3:**
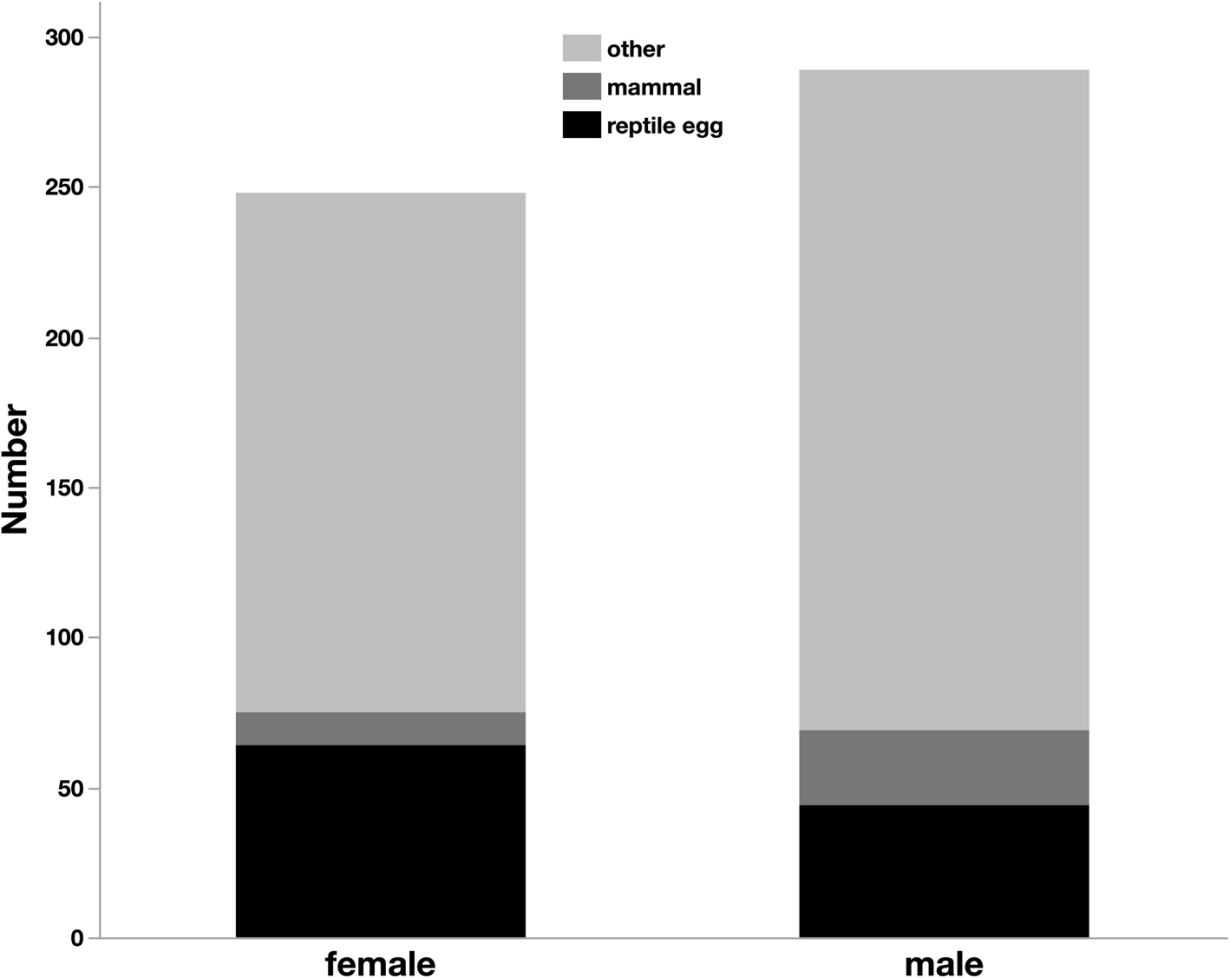
Comparison of prey items recorded in the faeces of female and male Slatey-Grey Snakes over the body-size range common to both sexes (800–1230 mm snout-vent length). Reptile eggshells were more common prey items of female snakes than of males. Values are percentage representations from 248 faecal samples from females and 289 from males

We obtained a total of 837 faecal samples from 530 individual Slatey-Grey Snakes between 1998 and 2021. One hundred and sixty-three of the samples contained reptile eggshells, 43 contained mammal hair and the rest consisted mainly of unidentifiable prey (likely anurans). Adult males and females differed in the types of prey (*df* = 1, *χ*^2^ = 4.45, *P* = 0.035). Females were more likely than males to have reptile eggshells in their faeces (23.6% *versus* 16.7%) but had similar amounts of mammal hair (3.9% *versus* 5.9%) and unidentified (mostly anuran) prey (72.4% *versus* 77.4%) (Fig. 3). Prey type was not significantly affected by snake SVL (*df* = 1, *χ*^2^ = 3.21, *P* = 0.073).

The majority of reptile eggs detected in Slatey-Grey Snake faeces were likely to be from Keelbacks, rather than some other egg-laying reptile. Eggs laid by other ground-nesting reptiles at our study site (agamid and scincid lizards) that are potential prey for Slatey-Grey Snakes are much smaller than Keelback eggs, and thus are easily distinguishable. Based on 201 average monthly counts, the number of Slatey-Grey Snake faeces containing reptile eggs increased with the number of gravid Keelbacks captured (Spearman *r* = 0.20, *P* = 0.013).

### Is the dietary divergence between sexes a by-product of sexual size dimorphism?

We obtained 537 faecal samples from Slatey-Grey Snakes in the size range that males and females overlapped (800–1230 mm SVL). Amongst this restricted sample, females were still significantly more likely than males to have reptile eggshells in their faeces (25.8% *versus* 15.2%, *df* = 1, *χ*^2^ = 4.35, *P* = 0.0370, Fig. 3). Prey type was again not significantly affected by snake SVL (*df* = 1, *χ*^2^ = 0.87, *P* = 0.352).

In gape-limited species where the sexes differ in relative head size, jaw dimensions might also affect diet composition. At the range of body sizes where adult male and female Slatey-Grey Snakes overlap, females have larger jaw lengths relative to SVL than do males (*F*_1,1911_ = 10.56, *P* = 0.001). The magnitude of that divergence is trivial however. Based on the prediction equations from our ANCOVA, for an individual Slatey-Grey Snake with a SVL of 1000 mm, predicted jaw length is 28.69 mm for a female and 28.54 mm for a male.

### Is the dietary divergence between the sexes related to differential foraging or activity patterns in males and females?

**Fig. 4:**
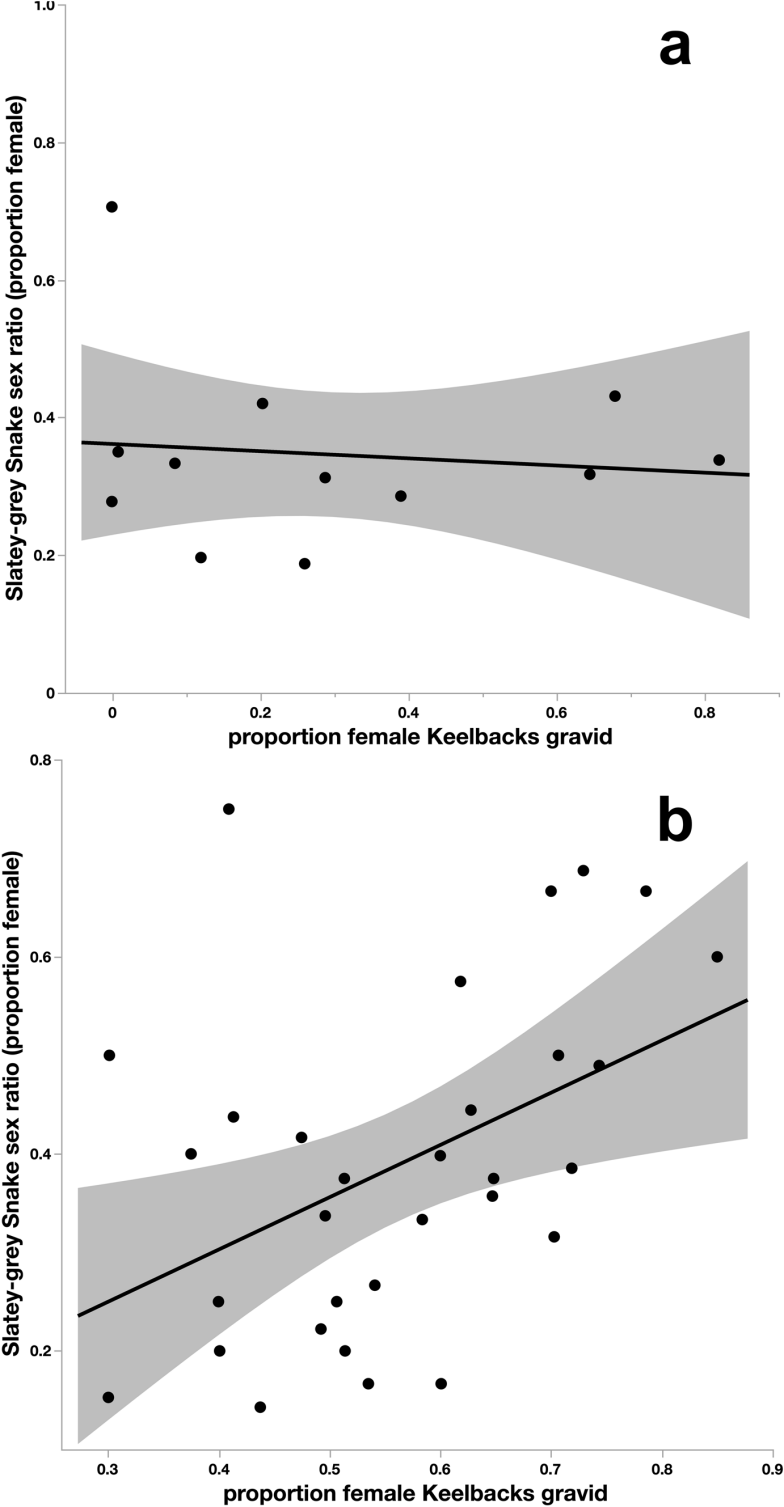
Temporal comparison of average sex ratios (proportion female) of adult Slatey-Grey Snakes captured per month relative to the average proportion of adult female Keelback Snakes captured in those same months (**a**). Spatial comparison of average sex ratios (proportion female) of adult Slatey-Grey Snakes captured in each of 31 50-m segments of the Fogg Dam wall relative to the average proportion of adult female Keelback Snakes captured in those same 50-m segments (**b**). Shaded areas indicate the 95% confidence interval around the fitted lines

Average monthly sex ratios of adult Slatey-Grey Snake captures were not significantly related to the average proportion of female Keelbacks that were gravid (*R*^2^ = 0.01, *F*_1,10_ = 0.12, *P* = 0.73; Fig. 4). Thus, female Slatey-Grey Snakes were not more common or more active than males during Keelback nesting periods. However, during the months when Keelbacks were nesting (March–November), female Slatey-Grey Snakes were more likely to be captured in the vicinity of Keelback oviposition sites (*R*^2^ = 0.20, *F*_1,29_ = 7.15, *P* = 0.0122; Fig. 4).

### Do female Slatey-Grey Snakes derive more benefit from eating reptile eggs than do males?

Our analyses on the impact of reptile-egg availability, temperature and rainfall on reproductive output and body condition of Slatey-Grey Snakes revealed the following patterns.

**Fig. 5:**
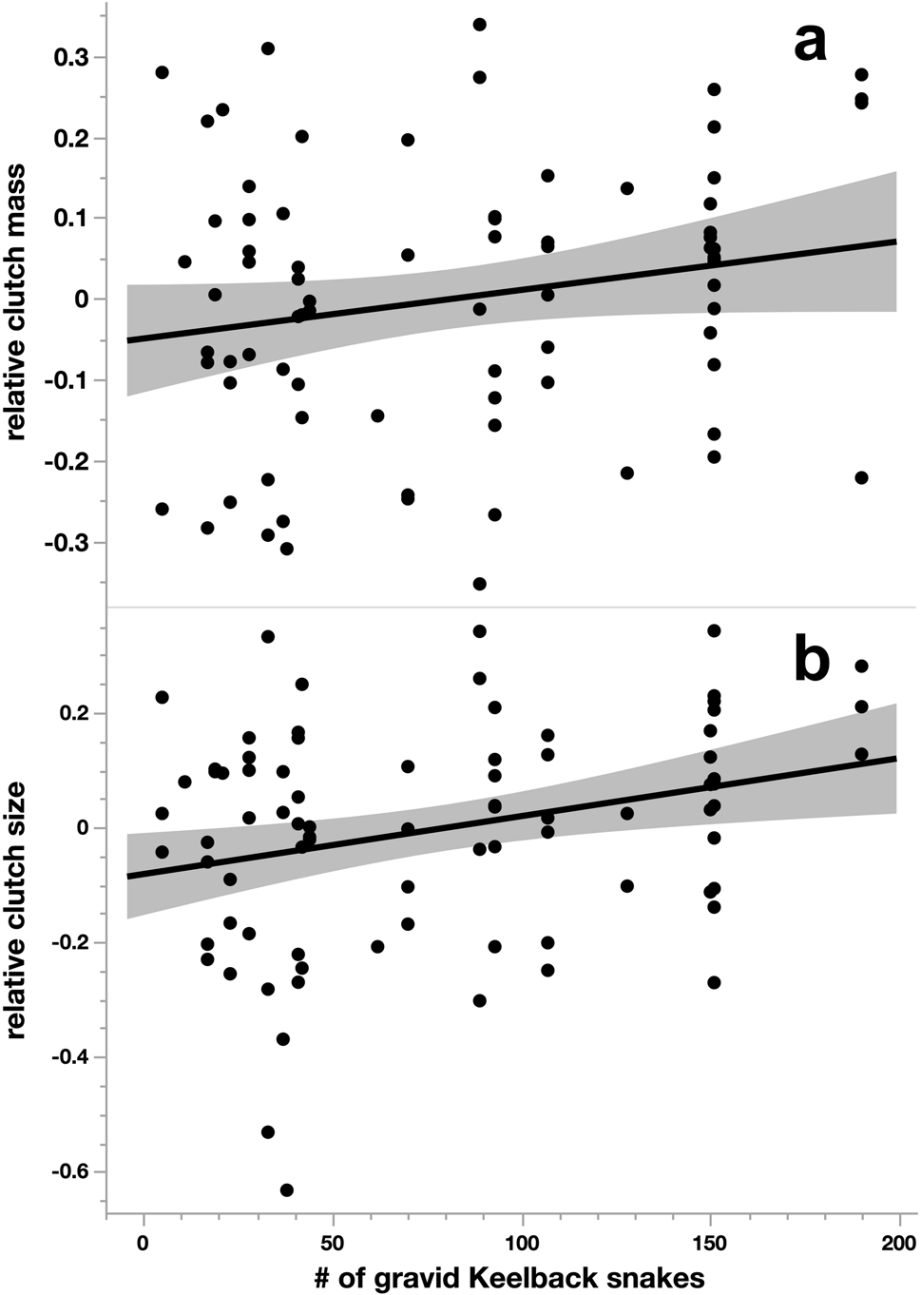
Effects of prey abundance on reproductive traits of female Slatey-Grey Snakes. Clutch mass (**a**) and clutch size (**b**) increased with availability of reptile eggs (indexed by numbers of gravid Keelback Snakes). Relative clutch mass and size are residuals from regressions on maternal snout-vent length to correct for body size effects for graphical purposes. Shaded areas indicate the 95% confidence interval around the fitted line

- *Frequency of reproduction* We captured 208 adult female Slatey-Grey Snakes (total of 366 captures) during the 20 reproductive seasons where we had data on prey types. We found 56 individual females gravid on a total of 69 occasions (0–50% of females gravid per year). All five measures of reproduction were positively related to female body size (all *P* ≤ 0.032; Table 1). Rates of reproduction were not significantly related to ambient temperature or rainfall nor to the availability of small mammals, frogs or reptile eggs (all *P* > 0.25; Table 1).
- *Body condition* Maternal body condition post-oviposition was not related to availability of any prey category or climate measure (all *P* > 0.06; Table 1). Body condition of similarly sized adult male snakes captured during each breeding season was also unrelated to any prey category or climate measure (all *P* > 0.21).
- *Reproductive output per clutch* Mean egg mass increased with maternal SVL but was not significantly linked to annual variation in any prey types or climatic measures (all *P* ≥ 0.146, Table 1). However, years with more nesting activity by Keelbacks saw higher mean values for clutch mass and clutch size in Slatey-Grey Snakes (both *P* ≤ 0.03, Table 1; Fig. 5). For perspective, based on the parameter estimates in Table 1, each increase of 50 gravid Keelbacks corresponded to an increase in Slatey-Grey Snake clutch mass of 4 g and an increase in clutch size of 0.8 eggs.

**Table 1:** Results of mixed-model analysis (with individual ID and breeding season year as random effects) on the effects of air temperature and prey abundances on reproductive traits of female Slatey-Grey Snakes

Female Slatey-Grey Snake trait	Effect	Estimate ± SE	*df*	*F*	*P*
Proportion female of gravid	SVL	0.0378 ± 0.018	1, 360	4.65	**0.0317**
Gravid	Mammals	−0.0021 ± 0.006	1,24	0.11	0.7378
	#gravid Keelbacks	0.0038 ± 0.006	1, 14	0.46	0.5094
	Frogs	0.2615 ± 0.462	1, 15	0.32	0.5798
	Temperature	0.4461 ± 0.389	1, 39	1.32	0.2581
	Rainfall	0.0008 ± 0.001	1, 31	0.51	0.4826
Log post-laying	SVL	0.0339 0.002	1, 61	212.24	**< 0.0001**
Body mass (g)	Mammals	−0.0008 ± 0.001	1, 43	1.63	0.2083
	#gravid Keelbacks	0.0007 ± 0.001	1, 37	1.81	0.1867
	Frogs	−0.0167 ± 0.034	1, 33	0.25	0.6233
	Temperature	0.0797 ± 1.041	1, 23	3.81	0.0635
	Rainfall	0.0002 ± 0.000	1, 25	2.14	0.1560
Mean egg mass (g)	SVL	0.0506 ± 0.016	1, 59	9.63	**0.0029**
	Mammals	0.0080 ± 0.006	1, 14	1.87	0.1934
	#gravid Keelbacks	−0.0088 ± 0.005	1, 14	3.27	0.0922
	Frogs	0.0390 ± 0.353	1, 7	0.01	0.9154
	Temperature	−0.2624 ± 0.452	1, 12	0.34	0.5718
	Rainfall	−0.0008 ± 0.001	1, 12	0.37	0.5567
Clutch mass (g)	SVL	1.7132 ± 0.204	1, 59	70.55	**< 0.0001**
	Mammals	−0.0393 ± 0.056	1, 59	0.49	0.4849
	#gravid Keelbacks	0.0795 ± 0.036	1, 59	4.96	**0.0298**
	Frogs	−1.6024 ± 3.381	1, 59	0.22	0.6373
	Temperature	1.5957 ± 3.424	1, 59	0.22	0.6429
	Rainfall	0.0088 ± 0.010	1, 59	0.72	0.3982
Clutch size	SVL	0.1769 ± 0.028	1, 61	40.47	**< 0.0001**
	Mammals	−0.0086 ± 0.008	1, 61	1.25	0.2676
	#gravid Keelbacks	0.0160 ± 0.005	1, 61	10.38	**0.0020**
	Frogs	−0.1999 ± 0.462	1, 61	0.19	0.6668
	Temperature	0.3289 ± 0.475	1, 61	0.48	0.4908
	Rainfall	0.0014 ± 0.001	1, 61	1.03	0.3147

Bold type indicates values of *P* < 0.05

*SVL* snout-vent length, *“mammals”* # of rats and mice trapped per year, *“#gravid”* # of gravid Keelback snakes captured per year, *“frogs”* # of frogs counted in the study area each year, *“temperature”* mean daily temperature in that year, *“rainfall”* sum of rainfall in that year

## Discussion

The answer to our first question (see Introduction) is clear: our long-term ecological study shows that reptile eggs are eaten more frequently by adult female Slatey-Grey Snakes than by conspecific males (26 *versus* 15% of meals, respectively). This result cannot be explained by gape limitation, because head dimensions relative to body length are similar between the sexes, and the largest available eggs (those of Keelback Snakes) are eaten by even the smallest adult female Slatey-Grey Snakes. Thus, the sex divergence in diet is not attributable to sexual dimorphism in body size or relative head size, contrary to our second hypothesis. Instead, female Slatey-Grey Snakes are more common around Keelback nesting sites when eggs are being laid. Although this evidence is indirect, it suggests that female Slatey-Grey Snakes forage in these sites more than do conspecific males. One plausible reason for that behavioural shift is that the benefit from eating reptile eggs may be greater for female Slatey-Grey Snakes than for males. In terms of our fourth question, we found that in years with higher availability of Keelback eggs, female Slatey-Grey Snakes had higher reproductive output (clutch size and clutch mass). Body condition of male Slatey-Grey Snakes was not significantly linked to annual variation in Keelback nesting activity, suggesting that they may not exploit Keelback eggs to as great an extent as do females. Males instead appear to focus more on more common or readily accessible prey types (e.g. frogs) in the ‘other’ diet category.

The sex disparity in mean adult body sizes is greater in Slatey-Grey Snakes than in most other snake species (males average around 15% longer in SVL: Brown et al. 2017), driven by sexual selection for larger body size in males (Dubey et al. 2009). Given a positive interspecific correlation between SSD and dietary divergence between the sexes (Bauld et al. 2022), we might have expected sex differences in the diet of Slatey-Grey Snakes to be a consequence of the SSD in this species. That explanation also fits well with many cases of sexual divergence in diet in snakes. In species with a massive disparity in mean adult body sizes between the sexes, individuals of the smaller sex are physically incapable of ingesting a prey type that is frequently consumed by individuals of the larger sex. For example, adult female Carpet Pythons (*Morelia imbricata*) from one island population subsist primarily on Tammar Wallabies (*Macropus eugenii*) weighing around 3 kg, whereas conspecific male pythons feed mostly on House Mice (*Mus domesticus*, < 15 g: Pearson et al. 2002). Likewise, the fishes consumed by large-headed female Arafura Filesnakes (*Acrochordus arafurae*) are much larger than those taken by small-headed males of this species (Houston and Shine 1993). Because the prey types in these examples are too large to be ingestible by the smaller sex, the sex divergence in diets is plausibly attributed to the indirect effects of SSD combined with gape limitation (Houston and Shine 1993; Pearson et al. 2002).

In our study system, in contrast, the prey type differentially consumed by the sexes consists of relatively small slender prey items. Although the total nutritional content of a clutch of Keelback eggs is large, the clutch can be swallowed as a series of separate items (eggs) rather than as one large bulky item. Gape limitation, thus, is less important than it would be if each meal consisted of a single large prey item. Because female Keelbacks nest communally on restricted dry-soil sites on the dam wall (Brown and Shine 2002, 2009), a foraging Slatey-Grey Snake likely could encounter multiple nests in a single night’s searching. Thus, Keelback eggs offer an extreme case of multiple relatively small (and thus, easily ingestible items) within a single “meal”.

Several authors have suggested that dietary divergence between the sexes may also be driven by advantages of reducing ecological competition between males and females. For example, a pair of breeding birds may most efficiently utilise prey resources within their breeding areas if one sex forages for a different prey type than does the other (e.g. Phillips et al. 2011; Slagsvold and Sonerud 2007; Sonerud et al. 2014; Weimerskirch et al. 2014; Zalewski 2007). The argument for competition reduction is less compelling for taxa such as snakes, which lack pair-based territoriality (e.g. Huang et al. 2011).

More generally, sex-based dietary divergence in snakes may be driven by multiple processes. One common situation is that the importance of some large-bodied prey type correlates with snake body size, and that males and females overlap considerably but not totally both in body sizes and diets (e.g. Filippi et al. 2005; Ford and Hampton 2009; Loaiza-Lange et al. 2023; Luiselli et al. 2007; Shetty and Shine 2002; Vincent et al. 2004). In such cases, it is difficult to tease apart the roles of sex divergences in mean body sizes (i.e. SSD), in other morphological traits (e.g. relative head size) or in ecological traits (e.g. habitat use), *versus* sex-specific differences in optimal diet composition. These mechanisms are not incompatible, and it is easy to envisage a situation where they interact. For example, the largest individuals (and thus, mostly of one sex) may be the only ones that are able to physically overpower and ingest some large prey type, but diets also may diverge within the common size range of males and females because of other reproduction-related ecological factors. For example, a mate-searching male that travels extensively in search of females may encounter and consume a different array of prey types than would a sedentary female, especially in a species that utilises ambush predation. Similarly, anorexia during mate searching (in males) and during pregnancy (in females) may generate seasonal divergences between the sexes in feeding activity—and hence in diets, in a system where different types of prey are available at different times of year (Goiran et al. 2013; Shine and Goiran 2021). In Slatey-Grey Snakes, mating appears to occur during August–October, after the peak periods of Keelback nesting. Thus, testosterone-fuelled focus on mate searching is unlikely to explain the under-representation of reptile eggs in the diets of male Slatey-Grey Snakes during the Keelback nesting season. Instead, we speculate that males may simply utilise a more widespread and abundant prey base, such as frogs, rather than searching for rarer though potentially more nutritious reptile eggs.

We have no data to clarify why eating eggs had more benefit for female Slatey-Grey Snakes than for males, but intuition suggests a plausible answer. Producing a clutch of eggs requires very high inputs of energy and nutrients, for the eggshell (e.g. calcium) as well as for the yolk and associated components that must sustain embryonic growth and metabolism (Thompson and Speake 2002; Van Dyke et al. 2012). Given broad interspecific similarities in the chemical composition of eggs and eggshells (Fu 1999; Kölle et al. 2022), the nutrients available in the egg of another colubroid snake species likely are similar to those needed for a female Slatey-Grey Snake’s own clutch. The optimal nutrient mix for a male Slatey-Grey Snake, in contrast, may be the combination that most effectively fuels growth and the development of musculature (Bonnet et al. 1998), and hence consumption from other vertebrates may satisfy this criterion. As mentioned above, this explanation is consistent with other studies suggesting that specific nutrient-rich food types may enhance fitness in one sex more than the other (e.g. Beier 1987; Du Toit 2005; Houslay et al. 2015; Raubenheimer and Simpson 2018; Reddiex et al. 2013).

Future studies could usefully explore the proximate mechanisms by which female Slatey-Grey Snakes increase their consumption of Keelback eggs. Females might actively search out Keelback nests, using visual, olfactory or vomeronasal cues; or (as suggested by our data) might restrict their foraging to the sites at which Keelback oviposition predictably occurs (Brown et al. 2002). More generally, wildlife populations with strong allometric or sex-based divergences in prey types offer excellent model systems in which to identify morphological or behavioural modifications that affect dietary composition (e.g. Hagman et al. 2008; Vincent et al. 2004). Some cases undoubtedly reflect sex-specific benefits to specific prey types; for example, the piercing mouthparts of blood-feeding female mosquitoes can only be interpreted in this way. But the generality of that mechanism remains unclear. If evolution has favoured adaptive divergence in diets between the sexes, we might expect spatial and/or temporal variation in the female-preferred food type to be associated with variation in female reproductive output in taxa with sex divergences in trophic structures (such as herbivorous mammals and megacephalic turtles: Reddiex et al. 2013; Shine 1989).

Identifying causal influences that underly dietary variation is a challenging task, but cases of dietary divergence between the sexes offer a powerful research opportunity because we can compare individuals that are broadly similar in most aspects of morphology and ecology yet differ in diets. Future studies could identify study systems in which some relevant factors do not apply (e.g. a species that lacks SSD; or a prey type whose consumption is not constrained by gape limitation), and then investigate whether intrapopulation divergences in the adaptive value of a specific prey type drive rates of consumption of that prey.

## Supplementary Information

Below is the link to the electronic supplementary material.

Supplementary file1 (XLSX 91 KB)

## Data Availability

Data have been included as a Supplementary File.

## Code availability

NA.
